# Increased network centrality of the anterior insula in early abstinence from alcohol

**DOI:** 10.1111/adb.13096

**Published:** 2021-08-31

**Authors:** Cecile Bordier, Georg Weil, Patrick Bach, Giulia Scuppa, Carlo Nicolini, Giulia Forcellini, Ursula Pérez‐Ramirez, David Moratal, Santiago Canals, Sabine Hoffmann, Derik Hermann, Sabine Vollstädt‐Klein, Falk Kiefer, Peter Kirsch, Wolfgang H. Sommer, Angelo Bifone

**Affiliations:** ^1^ Center for Neuroscience and Cognitive Systems Istituto Italiano di Tecnologia Rovereto Italy; ^2^ Univ. Lille, Inserm, CHU Lille, U1172 ‐ LilNCog ‐ Lille Neuroscience & Cognition Lille France; ^3^ Department of Addictive Behavior and Addiction Medicine, Central Institute of Mental Health, Medical Faculty Mannheim University of Heidelberg Mannheim Germany; ^4^ Center for Biomaterials and Tissue Engineering Universitat Politècnica de València Valencia Spain; ^5^ Instituto de Neurociencias Consejo Superior de Investigaciones Científicas and Universidad Miguel Hernández San Juan de Alicante Spain; ^6^ Department for Clinical Psychology, Central Institute of Mental Health, Medical Faculty Mannheim University of Heidelberg Mannheim Germany; ^7^ Institute of Psychopharmacology, Central Institute of Mental Health, Medical Faculty Mannheim University of Heidelberg Mannheim Germany; ^8^ Center for Mind/Brain Sciences University of Trento Trento Italy; ^9^ Department of Molecular Biotechnology and Health Sciences University of Torino Torino Italy

**Keywords:** alcohol use disorder, functional connectivity, insula, naltrexone, resting‐state fMRI

## Abstract

Abnormal resting‐state functional connectivity, as measured by functional magnetic resonance imaging (MRI), has been reported in alcohol use disorders (AUD), but findings are so far inconsistent. Here, we exploited recent developments in graph‐theoretical analyses, enabling improved resolution and fine‐grained representation of brain networks, to investigate functional connectivity in 35 recently detoxified alcohol dependent patients versus 34 healthy controls. Specifically, we focused on the modular organization, that is, the presence of tightly connected substructures within a network, and on the identification of brain regions responsible for network integration using an unbiased approach based on a large‐scale network composed of more than 600 a priori defined nodes. We found significant reductions in global connectivity and region‐specific disruption in the network topology in patients compared with controls. Specifically, the basal brain and the insular–supramarginal cortices, which form tightly coupled modules in healthy subjects, were fragmented in patients. Further, patients showed a strong increase in the centrality of the anterior insula, which exhibited stronger connectivity to distal cortical regions and weaker connectivity to the posterior insula. Anterior insula centrality, a measure of the integrative role of a region, was significantly associated with increased risk of relapse. Exploratory analysis suggests partial recovery of modular structure and insular connectivity in patients after 2 weeks. These findings support the hypothesis that, at least during the early stages of abstinence, the anterior insula may drive exaggerated integration of interoceptive states in AUD patients with possible consequences for decision making and emotional states and that functional connectivity is dynamically changing during treatment.

## Graphical abstract

Network analysis of brain functional connectivity in AUD patients indicates that the anterior insula plays an exaggerated integrative role during early abstinence. Prolonged abstinence and treatment partially normalize connectivity of the insula.

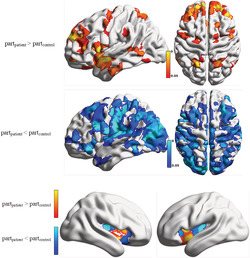

## 1 INTRODUCTION

Alcohol use disorder (AUD)—also called ‘alcoholism’—is among the most prevalent and severe psychiatric conditions worldwide,^1^ with many affected individuals drinking at levels that result in life‐threatening conditions.^2^ Neurobiological mechanisms driving long‐term adaptive and degenerative changes during alcohol abuse and subsequent recovery are poorly understood and continue to be of scientific interest.^3^ Current neurobiological constructs to explain the loss of control over and compulsive urges for drinking posit both impaired executive control over behaviour and sensitized responses to bottom‐up signals from emotional and motivational input that are largely mediated by brain circuits involved in reward and stress processing.^4^ Accordingly, an addiction circuitry is proposed, whereby brain regions that have received particular attention in AUD include structures at the basal forebrain such as ventral striatum and amygdala, as well as prefrontocortical areas involving anterior cingulate, medial prefrontal and insular cortices. However, although most studies have focused on the role of distinct brain regions, little is known about their functional interactions on a network level.

An overall view on brain activity and identification of large‐scale functional connectivity networks can be obtained from resting‐state functional magnetic resonance imaging (rsfMRI). This method assesses spontaneous fluctuations in the blood oxygen level dependent (BOLD) signal that occur in resting individuals and that show temporal correlations across functionally related brain areas. Initial studies of global resting‐state connectivity in AUD patients used probabilistic independent component analysis (ICA) to identify common large‐scale networks and compared functional connectivity between alcoholic patients and healthy controls.^5^, ^6^, ^7^, ^8^, ^9^, ^10^, ^11^, ^12^, ^13^, ^14^ These studies showed overall integrity of large‐scale functional networks in patients, with some alterations in connectivity within main networks. A few studies reported increased connectivity within prefrontal and frontobasal networks including default, salience and executive networks in patients^5^, ^7^, ^9^ or young binge drinkers.^8^, ^11^ Others found weaker intrinsic connectivity within these networks.^6^, ^12^, ^13^, ^14^ These discrepancies may be the result of different states of the patients enrolled in the various studies, or of differences in the methods used to compute functional connectivity. Hence, a number of important questions are still open.

Investigations on the functional role of brain networks in health and disease are dependent on an understanding of their organizational principles under these conditions. To this end, a powerful framework is provided by graph‐theoretical methods based on an explicit network representation of functional connectivity. In graph analysis, anatomically defined brain regions are treated as nodes of the network, and interregional correlations of spontaneous fluctuations in BOLD signal denote the edges. Of particular interest is the modular structure of brain networks, that is, the presence of subsets, or clusters, of nodes that are more densely connected among themselves than to nodes in other modules. This feature provides a measure of the balance between functional segregation and integration in the brain and is critical to interpret and classify the role of nodes within the topology of the network. Indeed, highly connected nodes, or hubs, that connect several different modules are dubbed connector hubs and are responsible for the integration of the entire network. Growing evidence indicates that connector hubs, characterized by high topological centrality and connectivity, are particularly vulnerable and may be implicated in neuropsychiatric disorders.^15^

Several methods have been proposed to resolve the modular structure of complex networks.^16^ The most popular approach is Newman's ‘modularity maximization’^17^ and variations thereof. A few large modules, including default mode and sensorimotor, occipital and frontoparietal networks, have been found using Newman's method with remarkable consistency.^18^ However, differences in the brain modular organization between healthy subjects and psychiatric patients have proven hard to demonstrate conclusively.^10^ This lack in sensitivity may be due to important limitations in Newman's approach. Recently, we have demonstrated the deleterious effects of these shortcomings, including a resolution limit that prevents detection of modules that are smaller than a scale determined by the size of the entire network.^19^, ^20^ Moreover, we have shown that these limitations can be overcome by recent approaches based on graph information theory,^21^, ^22^, ^23^ thus providing sharper tools to assess the modular organization of functional connectivity networks^19^, ^20^ and to apply advanced network statistics for comparing experimental conditions.^24^

Here, we leverage these important methodological advances to identify topological differences in large‐scale brain networks (>600 a priori defined nodes^25^) of AUD patients and healthy controls. rsfMRI data were obtained from a recently published clinical trial in treatment‐seeking patients that after a baseline assessment offered add‐on treatment with the approved anticraving medication naltrexone (NTX) in a naturalistic, longitudinal open‐label design.^26^, ^27^, ^28^ In this data set, we explore the stability and potential susceptibility to therapeutic intervention of resting‐state networks in AUD patients.

## 2 METHODS AND MATERIALS

### 2.1 Participants

The experimental groups consisted of 35 males, recently detoxified, abstinent alcoholics (age 45 ± 9, abstinence days 21 ± 7, 260 ± 120 [g]/day of alcohol pretreatment) and 34 healthy male volunteers (age 41 ± 10) recruited within the ERA‐NET NEURON TRANSCALC study (WHO International Clinical Trials Registry Platform: DRKS00003357). Analyses of task‐based fMRI and diffusion data from this trial have been recently reported.^26^, ^27^, ^28^ Here, we focus on the investigation of *functional connectivity* from rsfMRI data and hence included a subset of participants of the previously reported sample for whom high‐quality resting‐state data was available. Clinical characteristics are listed in Table S1. The key inclusion criteria for the AUD group were the diagnosis of an alcohol dependence according to DSM‐IV (here equated to AUD), controlled abstinence of at least 2 weeks prior to the MRI session and completion of medically supervised detoxification (treatment of withdrawal symptoms with short‐acting benzodiazepines had to be completed for at least 3 days). Patients with psychiatric comorbidities or abuse of other substances (except smoking) were excluded. Smokers were allowed to smoke ad libitum during the study. Patients participated in a standardized inpatient multi‐professional medically‐supervised therapy schedule—here termed intensive withdrawal treatment (IWT) (see Loeber et al.^29^). After baseline assessment, patients were offered the choice between treatment as usual, treatment that was continued IWT, and IWT plus adjuvant oral NTX (50 mg per day) in a naturalistic open‐label free‐choice design. A follow‐up fMRI scan was scheduled for all patients 2 weeks into treatment with either NTX plus treatment as usual or ITW only (M = 15.7 days, SD = 3.5). For 29 patients, two complete rsfMRI data sets were available, with 17 subjects receiving IWT + NTX and 12 IWT only (Table S2). Details on the clinical assessment of patients are reported in the Supporting Information, Extended Methods section. The study was approved by the local ethics committee in accordance with the Declaration of Helsinki.^30^

### 2.2 MRI acquisition and preprocessing

rsfMRI data were part of a multimodal assessment protocol^26^ and collected with a 3T whole‐body magnetic resonance (MR) scanner (MAGNETOM Trio with TIM technology; Siemens, Erlangen, Germany) using echo‐planar imaging (EPI) and simultaneous acquisition of physiological data. Details of the MRI protocol are reported in the Extended Methods section of the Supporting Information. In short, the fMRI data were band‐pass filtered in the frequency range 0.01–0.1 Hz and preprocessed using standard methods implemented in FSL and SPM (detailed in the Supporting Information). A multiple regression model was applied to remove head‐motion (see Supporting Information); physiological data (respiration, heartbeat) were acquired in‐scanner, and their effects were removed from the data using the Aztec toolbox (further details in the Supporting Information). Patients and controls were carefully matched on the basis of in‐scanner motion levels using framewise displacement (FD) and DVARS. Comparisons of motion parameters DVARS and FD for the experimental groups are reported in the Supporting Information (Tables S4 and S5, Figures S1 and S2). Sparsification of the resulting networks was also applied to further control potential residual in‐scanner motion (see below).^31^ Structural MR images were also acquired and analyzed within a voxel‐based morphometry (VBM) framework to assess potential differences in grey matter density and structure.

### 2.3 Connectivity graphs

Detailed information on the graph analysis methods and of the definitions is reported in the Supporting Information. Shortly, the template of Crossley et al.^25^ was used to parcellate the whole brain of each participant into 638 cortical and subcortical regions of interest, each representing a node in the network. This atlas, based on functional parcellation, has been previously applied to the study of other psychiatric disorders, for example, schizophrenia.^25^, ^32^ BOLD time series were extracted at the image voxel level and averaged over the voxels comprised in every node to compute node‐level timecourses; Pearson correlation coefficients were calculated for all pairs of nodes, thus providing an adjacency matrix for each subject in the study. Group‐level functional connectivity matrices were computed by Fisher‐transforming and subsequent averaging of individual's adjacency matrices and subsequently sparsified by percolation analysis.^33^ Sparsification procedures are often applied to remove the weakest edges, which are the most affected by experimental noise and likely to contain spurious correlations. We have recently shown that the percolation threshold maximizes information extracted by the subsequent application of community detection algorithms^24^ and applied and validated the method in human^21^ and animal^22^ studies, as well as in synthetic networks.^24^ Moreover, percolation analysis has been shown to effectively remove potentially spurious correlations (e.g., from residual in‐scanner motion) while preserving large‐scale structure of functional connectivity networks.^31^

### 2.4 Network metrics

From the adjacency matrix, we extracted the distribution of z‐score (corresponding to the weighted edges of our network), and we computed nodal and global measures of connectivity. The degree of a node represents the number of connection towards other nodes. The network density indicates the ratio between the connections in the matrix after sparsification and all possible connections.^34^ Global efficiency can be interpreted as a measure of how efficiently information is exchanged across the network.^35^ It is defined as the inverse of the harmonic mean of the shortest weighted path lengths connecting every pair of nodes and is inversely related to the network characteristic path length.^36^

Local efficiency is defined as the efficiency of a local subgraph consisting of a node *i*'s nearest neighbors, excluding the node *i* itself, and quantifies a network's resistance to removal of that node on a local scale. The definition of weighted local efficiency used in this manuscript is the one given by Rubinov et al.^36^ Further information on the computation of connectivity graphs and the definitions of network metrics are reported in the Supporting Information.

### 2.5 Modular organization by InfoMap

Here, we have applied InfoMap, a method based on the optimization of a cost function dubbed map equation.^37^, ^38^ A weighted version of InfoMap was applied to the sparsified networks. We have recently shown that this method is superior to Newman's modularity in terms of sensitivity and specificity in the presence of heterogeneously distributed modules and overcomes some of the fundamental limitations of Newman's modularity.^20^ Moreover, InfoMap has been widely applied and validated in community detection studies in natural networks, including brain connectivity networks from clinical studies involving neuropsychiatric patients.^39^ In order to generate a stable solution from a nondeterministic method like InfoMap, we applied a consensus approach.^40^

All visual representations of the anatomical distribution of modules and topological parameters were produced using the BrainNet Viewer toolbox^41^ and MRIcron.^42^

To evaluate edge‐level statistical differences between groups, the network‐based statistics (NBS) toolbox was used.^43^

### 2.6 Group‐level comparison of modular organization

The primary endpoint of this study is the assessment of significant differences in functional connectivity modular organization between detoxified patients and matched healthy controls. In a secondary exploratory analysis, we tested for within‐subject differences in a subgroup of patient who accepted treatment within an open‐label design by comparing scans at the beginning and the end of the additional two‐week period of abstinence.

Statistical comparison of modular organization between groups was performed by determining the community structure of each subject and by calculating the ‘distance’ between pairs of individuals as measured by normalized mutual information (NMI), an information‐based metric that captures the structural differences between two partitions, as proposed in Bloch et al.^23^ This statistical method is based on the idea that if variance in the community structure data is reliably explained by group membership, then the mean NMI between all possible pairs of participants within an experimental group should be higher than the mean NMI of pairs of participants from random groups. Because the distribution of group means NMI is not known a priori, a null distribution is generated through a permutation method between the two experimental groups (10 000 permutations). The *p*‐value was then defined as the number of times that the permuted group similarity exceeded the within‐group similarity (normalized by the number of permutations).

The same analysis was applied in the comparison between the pre‐ and post‐treatment resting‐state data in the group of patients and in the two subgroups (IWT only or IWT + NTX). Significance levels were FDR‐adjusted.

Additionally, we tested for differences in the overall connectivity strength by comparing edge‐weight distributions between experimental groups (by Student's *t*‐test).

### 2.7 Participation coefficient

To complete the investigation at the node level, we considered the alteration in node role between the two populations based on the differences in modular organization. To this end, we adopted Guimerà and Amaral's classification scheme,^44^ whereby nodes are classified by their within‐community degree (a measure of how well connected a node is to other nodes in the same community) and their participation coefficient *P*, a parameter that reflects the extent to which a node is connected to nodes in other modules. For a definition of *P*, see Supporting Information: Nodes with high participation coefficient are characterized by high centrality and are important for the integration of various modules into a cohesive, efficient network structure.^44^ Node‐wise participation coefficients were compared between experimental groups with a one‐tailed Student's *t*‐test, Bonferroni‐corrected.

### 2.8 Post hoc correlations with clinical variables

To test if clinical variables could predict alterations in global efficiency metrics or for selected participation coefficients (see results), we used multiple linear forced entry regression analysis (IBM SPSS Statistics software Version 20, IBM Corp., Armonk, NY, USA) with a model comprising alcohol consumption (standard drinks containing 12 g alcohol per drinking day) and severity of alcohol dependence (ADS) as well as age as control variable. Likewise, the effect of smoking was tested including the variables age, Fagerström Test for Nicotine Dependence (FTND) and pack‐years.

Follow‐up relapse data and neural connectivity data were available for 17 NTX patients and 10 patients receiving standard treatment (*n* = 27). Cox regression models were implemented to test the main effect of NTX on time to first severe relapse, as well as associations between relapse risk and local and global connectivity measures (i.e., global efficiency and local efficiency and participation coefficients of seven insular nodes) and the interaction of both factors.

## 3 RESULTS

### 3.1 Reduced overall connectivity in alcohol‐dependent patients

**FIGURE 1:**
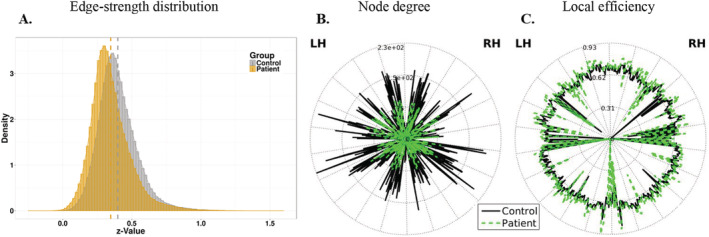
Reduced overall connectivity in AUD patients. (A) Z‐value distributions for the adjacency matrices of the two experimental groups; a left shift in the distribution from alcoholics denotes overall weaker connectivity in patients. (B) Degree (unweighted) of each node for the patient and control groups (in green and black, respectively). (C) Local efficiency value by nodes (same color scheme as in b). The nodes of the left and on the right hemisphere (LH and RH) are respectively on the right and on the left side of the circle

Global differences in functional connectivity were assessed by the edge‐weight distribution between patient and control groups (Figure 1A). A significant difference was observed in the average strength of the connections, indicating overall weaker connectivity in AUD patients (*t*‐test *p*‐value < 2.2 × 10^−16^). Further network parameters, namely, degree (unweighted) and local node efficiency, are compared in Figure 1B,C and Table S3. Although these parameters are not independent, the analyses reflect overall reduction in functional connectivity in the AUD group. Interestingly, the average of the local efficiency was similar for the two groups, suggesting that the resilience to node failure is preserved at a local level in AUD patients.^45^

### 3.2 Disruption of modular organization in alcoholics

**FIGURE 2:**
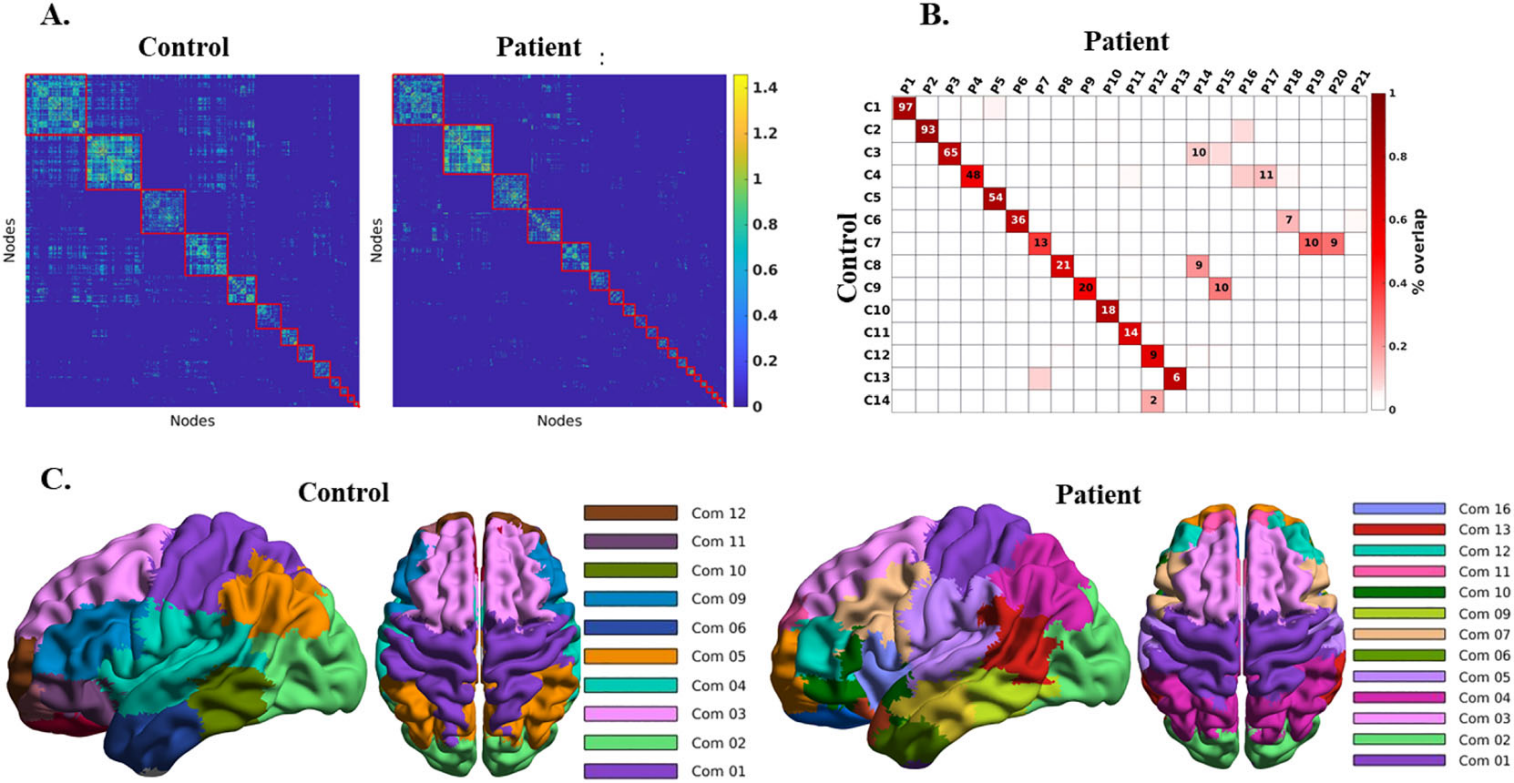
Comparison of adjacency matrices for patients and controls. (A) Group‐level adjacency matrices are shown with the node indexes rearranged by membership. The different modules are marked by red lines. (B) Matrix comparison with lines and columns corresponding to modules in the control and in the patient group, respectively, ordered by size with 1 indicating the largest community. The numbers in the cross‐elements of the matrix indicate the number of node overlaps between modules in the two groups. For example, Modules 1 and 2 are virtually identical in the two groups, whereas Module 7 of the control group (the basal module) corresponds to three different communities in the patient group, indicating that this community breaks apart in AUD patients. The colors of the cross elements refer to the modules displayed in (C), showing a cortical representation of the modular organization of functional connectivity for both groups. The colors denoting the communities were chosen independently in the two groups to maximize contrast between adjacent modules. This representation enables the identification of the anatomical districts comprised by the various communities. By way of example, Com1 of the control group includes mostly sensorimotor cortices and presents a closely corresponding module in the patient group. Com 2, which includes the visual cortices, is also consistent between groups. Com4 of the control group, consisting of supramarginal and temporal areas, is split into two sub‐modules (Com5 and Com16, respectively) in the patient group. A list with the anatomical description of all modules is reported in the Supporting Information

Figure 2A shows the group‐level adjacency matrices. NMI‐based non‐parametric permutation analysis^23^ showed significantly different community structures in patient and control groups (*p‐*value *= 0.029*, FDR‐corrected). We found 14 and 21 modules in the control and patient groups, respectively, the result of fragmentation of certain modules in alcoholics. To illustrate these differences, we calculated the overlaps between modules in the optimal partitions for the two groups by determining the number of common nodes within the modules (Figure 2B and Table S3). Interestingly, this analysis shows fragmentation of only a few modules in patients (see below) and other differences involving mostly singletons (single isolated nodes). The modular structure of connectivity in patients and controls is shown in Figure 2C.

NBS identified significantly weaker edges in patients versus controls (Figure S3). Widespread reduction in strength was observed in edges connecting different modules (off‐diagonal elements in the matrix of Figure S3). Conversely, differences in within‐module links are concentrated in a few regions, most prominently in the supramarginal and basal modules, which show more than 50% of the edges significantly weaker in patients than in controls. Hence, further analyses focused on these two subnetworks that show significant fragmentation in patients.

### 3.3 Fragmentation of basal and supramarginal modules in AUD patients

**FIGURE 3:**
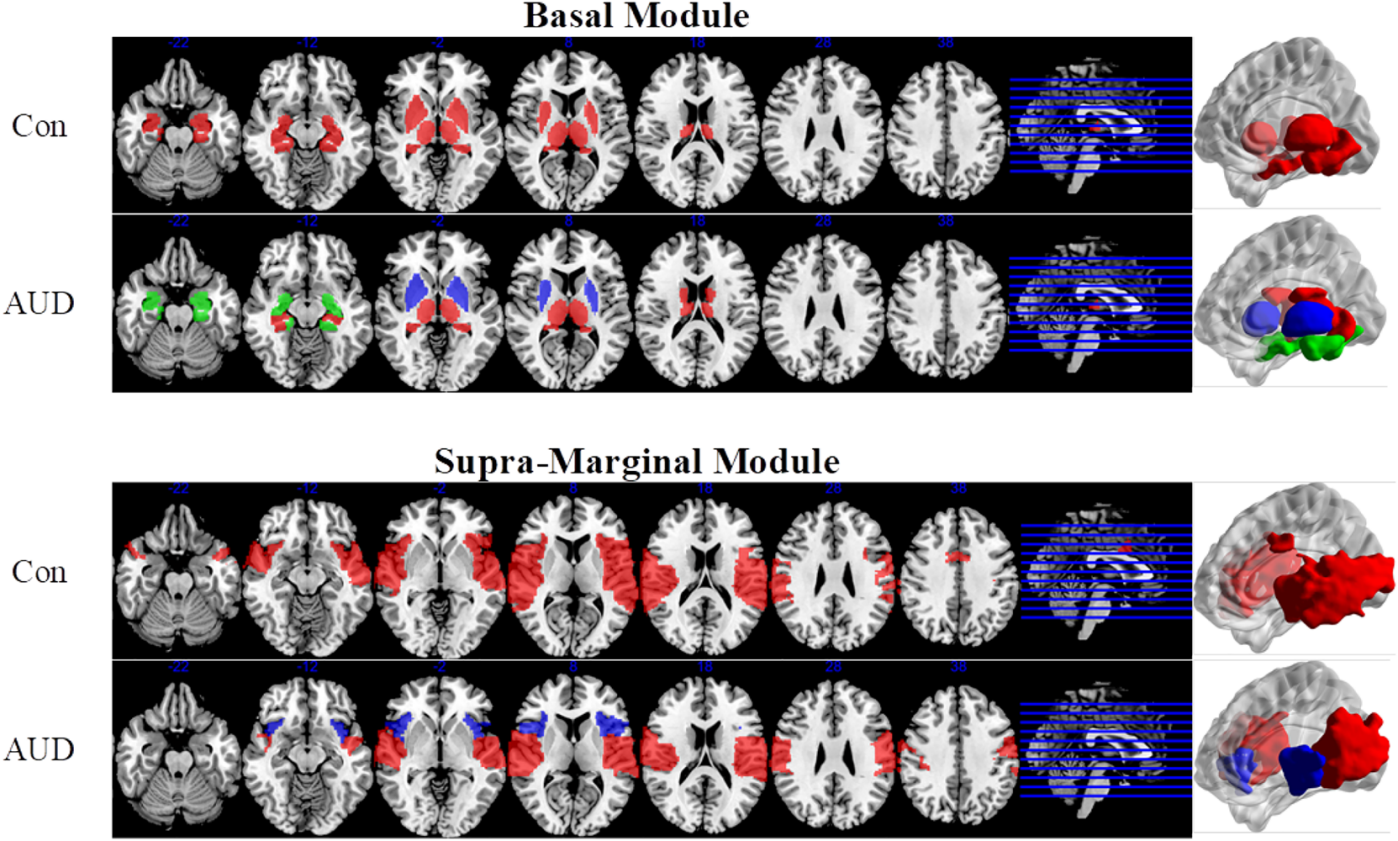
Fragmentation of the basal and supramarginal modules in AUD patients. Top panel: In the patient group, the basal module is subdivided into three communities including the amygdala, the caudate–thalamus and the pallidum‐putamen regions. Lower panel: The supramarginal‐temporal module in controls and patients, with a dissociation of the anterior part of the insula in the AUD group. These brain projections were created using to BrainNet Viewer^41^ and MRICron^42^

Figure 3 shows the organization of the basal and the supramarginal temporal functional subnetworks. The basal module, which includes the amygdala, pallidum, putamen, hippocampus, thalamus and caudate in the control group, is subdivided into three modules including amygdala, caudate–thalamus and pallidum–putamen in the patient group. The supramarginal temporal module found in controls shows dissociation of the anterior part of the insula forming an independent module in patients. Differences between groups in other modules are less pronounced, with occasional nodes in the superior parietal areas appearing as part of the somatosensory module of the control population, but not in patients, where they are joined with the precuneus and inferior parietal regions (Module 1, in purple, in Figure 2).

### 3.4 Increased centrality of anterior insula in AUD patients

**FIGURE 4:**
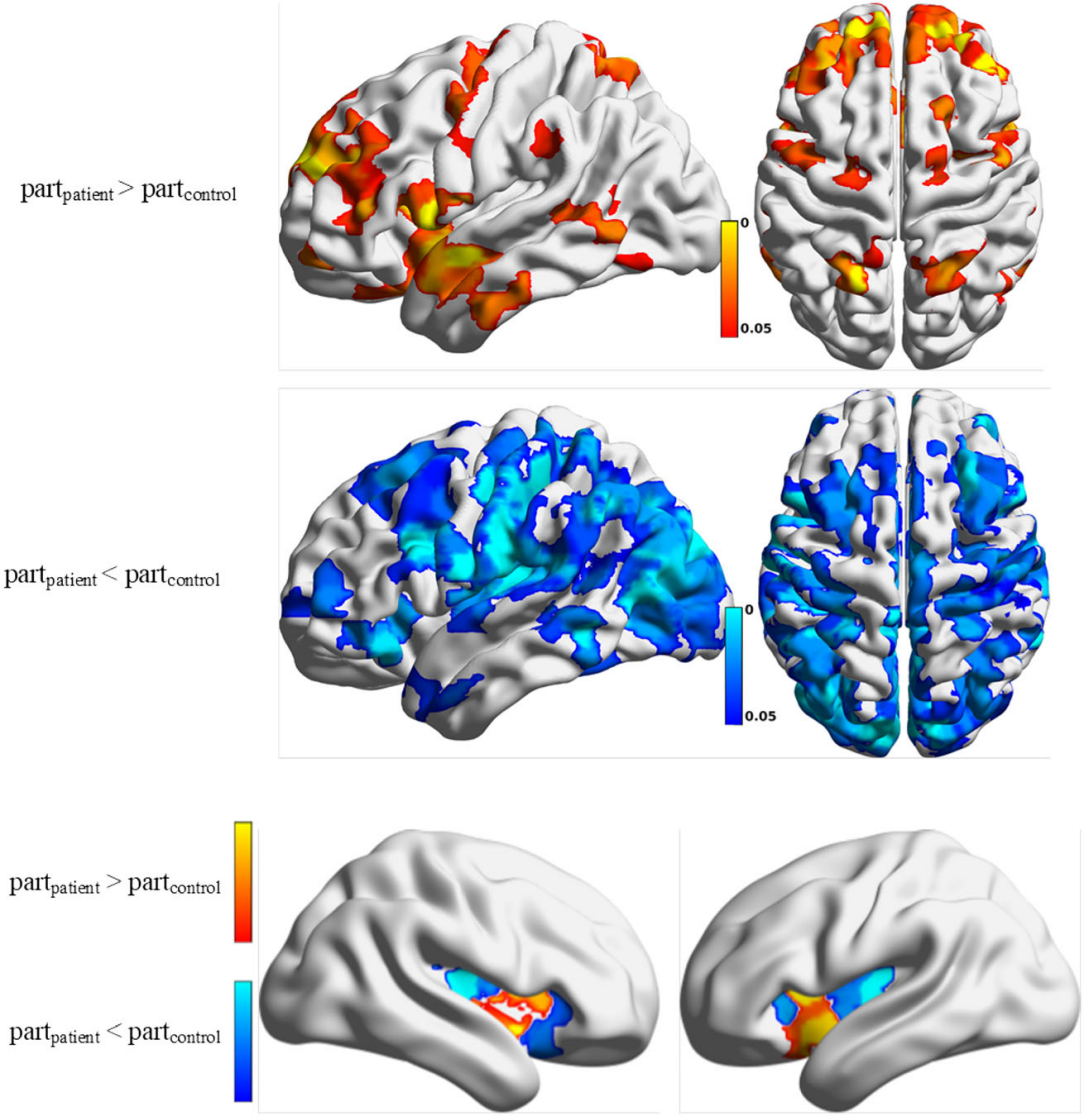
(A,B) Map of differences in participation coefficients between AUD patients and healthy controls. Projections of *p*‐values obtained by a one‐tailed Student's *t*‐test, Bonferroni‐corrected, are shown comparing node‐wise differences between groups, with the hypothesis of larger participation coefficient for the patients or for the controls in the top and bottom panels, respectively. (C) Differences in participation coefficients of the nodes in the insular cortex represented on an inflated brain template to expose the brain areas underneath the temporal lobes; *p*‐values obtained by a one‐tailed Student's *t*‐test, Bonferroni corrected. The anterior insula shows significantly increased centrality in patients, while participation coefficient in the posterior insula is reduced

Differences between node‐wise participation coefficient in patients and controls are displayed in Figure 4. Consistent with the generally decreased connectivity in AUD, the distribution of nodes with a significantly smaller participation coefficient in patients is more widespread and includes visual, sensory and auditory cortices, as well as parts of the middle frontal gyrus (Figure 4B). Surprisingly, despite the general decrease of functional connectivity in patients, some nodes present higher participation coefficient in patients than in controls. Large participation coefficients typically denote the connector hubs, that is, nodes with many connections pointing to different modules. Significant increases are detected in the frontal cortex and superior parietal areas (Figure 4A) and, most prominently, in the bilateral anterior insula (hidden in Figure 4A by the temporal lobe, shown in Figure 4C on an inflated brain template). Conversely, the posterior insula exhibits significant reduction in participation coefficient (Figure 4C).

### 3.5 VBM

A previous study demonstrated enlargement of the amygdala and a decrease of the insula volume in AUD patients.^46^ To rule out potential effects of alcohol‐induced morphometric differences between groups, we performed a VBM analysis. We found no evidence of significant morphometric differences between patients and controls in these regions (Figure S6), thereby excluding the potential cofounding effects of major morphometric alterations on functional connectivity assessments between groups.

### 3.6 Correlation with clinical variables

Exploratory regressions between age, clinical alcohol‐related variables (ADS and drinks per day during the last 90 days) and network measures revealed significant relationships only for the ADS in predicting the participation coefficient in the right anterior insula (Model *R* ^2^ = 0.18, ADS β = −0.48, *p* = 0.025). There were no significant findings in predicting global efficiency measures. Further, there was no effect of smoking variables. Contrary to our original publication,^26^ in the Cox regression, we did not find a main effect or interaction of NTX treatment on relapse risk (*p* > 0.05), which is likely due to the smaller size of the subsample used here. However, we found a significant effect of the participation coefficient of the left anterior insula that was associated with higher risk to relapse to heavy drinking (hazard ratio [HR] = 45.351, chi^2^ overall model = 6.159, *p* = 0.013), even when controlling for medication effects.

### 3.7 Effects of treatment on network properties

**FIGURE 5:**
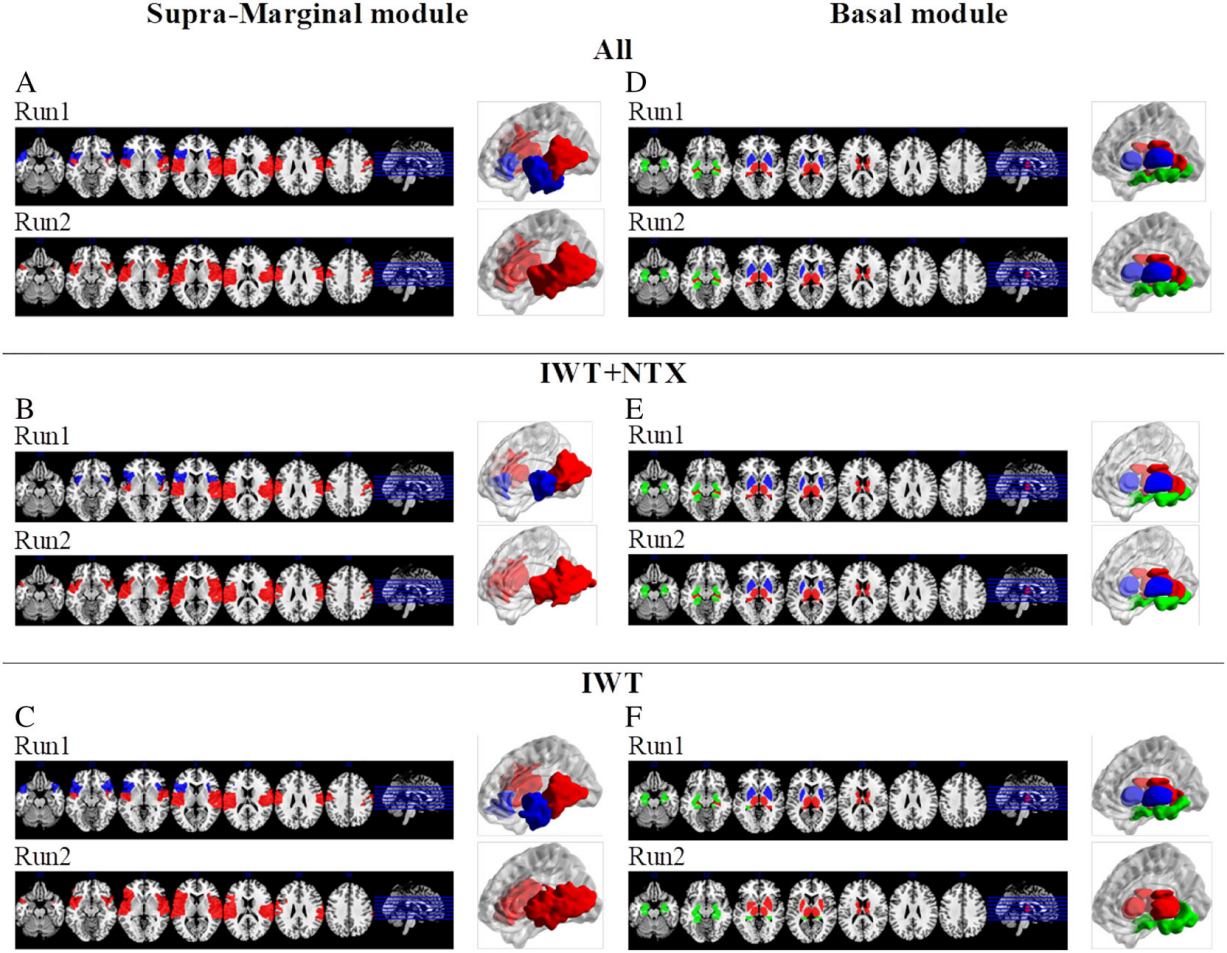
Effects of treatment on the basal and supramarginal modules in AUD patients. Left panel: (A) At a group level, all patients showed changes in the organization of the supramarginal module after 2 weeks of treatment, with a reversal of the dissociation of the anterior insula from the rest of the module; analysis of subjects treated with ITW + NTX (B) or ITW alone (C) showed that this effect occurs for both subgroups. Right panel: No effect of treatment was observed in the basal module, which remains subdivided after treatment at a group level (D) and for the ITW + NTX subgroup (E). A small effect was observed in the ITW(f) subgroup, with a few nodes of the caudate putamen changing membership after treatment

In the combined group of 29 patients participating in the naturalistic open‐label treatment, IWT alone or in combination with NTX, we observed a modest but significant increase in the global strength of functional connectivity, with a right shift of the edge weight histogram (Figure S4). Organization of the supramarginal and basal modules in patients at baseline and after 2 weeks of treatment is shown in Figure 5. Interestingly, the initial fragmentation of the supramarginal module was reversed, with the anterior insula reunited with the supramarginal nodes after 2 weeks of treatment (Figure 5A). The participation coefficient of the anterior insula showed significant reduction after 2 weeks (Figure S5). Conversely, the basal module organization remained fragmented over time (Figure 5D), and no significant effects on participation coefficient of the nodes included in this module were observed.

Analyses of the two subgroups of patients treated with IWT plus adjunct NTX (*n* = 17) or ITW alone (*n* = 12) showed very consistent results, with recovery of the supramarginal module in both cases (Figure 5B,C), and no significant effects in the basal module (Figure 5D,E).

## 4 DISCUSSION

Our graph‐theoretical analysis of rsfMRI functional connectivity revealed two main findings about the alterations in network organization in AUD patients compared with healthy controls. Firstly, in line with some previous studies,^6^, ^12^, ^13^, ^14^ we found a general decrease in functional connectivity in AUD patients as demonstrated by a highly significant reduction in the average strength of the connections. Secondly, on a local level, we found region‐specific alterations in the modular structure of functional connectivity. Specifically, fragmentation of the basal module in three smaller structures (caudate–thalamus, pallidum–putamen and amygdala–hippocampus) was observed in the alcoholic cohort. Moreover, within the supramarginal module, the insular cortex was found to break up in two different divisions in AUD patients, namely, a posterior and an anterior subdivision, in line with the well‐known functional and cytoarchitectural partition of the insula.^47^ This fragmentation was associated in the anterior insula with a significant increase in participation coefficient, a measure of the integrative role of a node within the connectivity network. These findings demonstrate that patients with a history of alcohol dependence show an alteration involving brain regions known to play a key role in addiction and provides a key to interpret the functional effects of disrupted connectivity in the alcohol‐dependent brain. The heterogeneous nature of the connectivity changes in AUD patients would have likely gone unnoticed by common functional connectivity analysis. Here, we took advantage of important methodological advances in graph‐based analyses of functional connectivity, finer network representation (>600 nodes), improved resolution^20^ and lower risk of motion‐related biases between different experimental groups.^24^, ^31^

Following the seminal work by Naqvi and Bechara,^48^ showing that damage to the insula was able to disrupt drug‐seeking behaviors in smokers, a number of studies highlighted a potential role of this region in drug addiction. For example, functional imaging studies showed that alcohol cues elicited greater activity within the insula than control stimuli and that this effect was stronger in AUD patients compared with healthy controls.^49^, ^50^ Moreover, reversible inactivation studies in rodents confirmed the critical role of the insula in mediating different aspects of drug addiction.^51^, ^52^, ^53^, ^54^, ^55^ The common thread of these studies is the idea that drug craving and cue‐triggered urges can be considered as a complex interoceptive state that is represented and mapped in the insular cortex, particularly in its anterior portion.^56^

Consistent with this idea, we observed an increased centrality of the anterior insula in AUD patients compared with controls indicating an exaggerated role of this region in the integration of interoceptive states into emotional and decision‐making processes in patients. Indeed, the anterior insula is part of the salience network that mediates attention and arousal as a result of external stimuli. Alterations in anterior insula connectivity, as described here, may underlie the attentional and cognitive bias towards drug and drug‐related cues often observed in addicted individuals.^57^, ^58^, ^59^ Interestingly, a significant association between participation coefficient in the left insula and risk of relapse was found even when controlling for treatment effects, thus suggesting that elevated participation coefficients may be predictive of vulnerability to relapse. Our findings support recent studies reporting increased connectivity of the insula in AUD patients.^13^, ^60^, ^61^ Interestingly, we found convergent results in a rat model of AUD.^62^ In that study, aberrant connectivity of the anterior insula was demonstrated in rats intermittently exposed to ethanol vapor, with weaker correlation between anterior insula and posterior insula and between the anterior insula and the cingulate cortex. Treatment with a D3 dopamine receptor antagonist, known to reduce alcohol consumption in animal models of AUD, resulted in partial recovery of functional connectivity as measured by rsfMRI.

Because the anterior insula also processes autonomic afferent input, it may be argued that changes observed in recently detoxified alcohol‐dependent patients may be caused by physiological confounds or bodily state variables (e.g., differences in heart rate and respiration) rather than a reorganization of connectivity at the brain network level. However, we note that physiological parameters, including heart rate and respiration, were measured during the fMRI experiment, with no significant differences between groups. Moreover, their effects were removed from the data in the preprocessing step, thus ruling out these variables as potential origin of the observed differences.

The effects of protracted abstinence was explored in patients who underwent a standardized therapy program with the option of adjuvant NTX in an open‐label naturalistic design,^26^ an opioid receptor antagonist with demonstrated albeit modest efficacy for relapse prevention.^63^ In the clinical trial, we found higher neuronal reactivity to alcohol cues in several brain regions in patients versus healthy controls. Cue reactivity increased over 2 weeks in the standard treatment group but not in the NTX group. NTX significantly attenuated alcohol cue reactivity in the left putamen and reduced relapse risk to heavy drinking within 3 months of treatment.^26^ Here, in a subpopulation of the original study, we observed a partial recovery of connectivity strength and of the structure of the supramarginal module. The participation coefficient of the insular cortex was significantly lower after 2 weeks of treatment. Importantly, these findings demonstrate that at least some alterations in functional connectivity observed in early withdrawal are actually reversible. Moreover, the observation of a reduction by treatment of the integrative role of the insula suggests a potential mechanism underlying amelioration of the condition.

Whether the trend towards recovery in the strength and structure of functional connectivity networks was driven by continued abstinence or reflected the psychoeducational or pharmacological intervention remains unclear from the present post hoc analyses of the two subgroups of patients receiving daily adjunct NTX or IWT only. Defragmentation of the supramarginal module was observed in both cases, thus suggesting that they are not related to the specific pharmacological mechanism of NTX, but rather reflect the change in the state of the condition during protracted abstinence. These effects appear to be driven by insular connectivity, because no significant changes were observed at the level of the basal module, the latter comprising dopaminergic pathways that are central to the brain reward system. A recent study by Morris et al.^64^ showed reduced connectivity in AUD patients, consistent with our results, and an effect of NTX on some topological parameters like local efficiency. We note that in that study, NTX was administered 2 h prior to the MRI scan and the modulation of connectivity reflected the acute effects of the drug. In our case, the effects of NTX were assessed at steady state after 2 weeks of daily treatment as adjuvant to IWT, which reflects clinical reality and more likely to represent chronic changes associated with amelioration of the condition.

Enlargement of the amygdala and a decrease of the insula volume have been demonstrated in severe cases of AUD compared with healthy controls.^46^ In our sample, we found no evidence of significant morphometric differences between groups in these regions. This is possibly due to the lower severity of the AUD in the subjects included in the present study (ADS average score [SD]: 14.08 [6.55] vs. 22.8 [6.1] in Senatorov et al.^46^). Time of abstinence before the scan was not different between the two studies, ruling out recovery related effects. On the other hand, the lack of major morphometric difference between patients and controls argues against the possibility that the observed differences in functional connectivity may result from regional misregistration of patient images onto a common template.

A recent study^65^ has investigated the effects of repeated deep transcranial stimulation of the insula in alcohol‐dependent subjects in a randomized control trial. Seed‐region analysis of resting‐state connectivity showed some effects of rTMS on insula connectivity, but reduction of craving scores and alcohol‐consumption measures were observed in both the sham and treatment group; alcohol use was resumed by both groups in the follow‐up period (12 weeks). Although this first attempt to target the insular cortex of alcohol‐dependent patients did not show clinical efficacy, it should be noted that transcranial magnetic stimulation (TMS) of a deep brain regions, like the insula, remains challenging, with a broad and intense involvement of more superficial and surrounding regions. Our study suggests that specific targeting of the anterior insula should be attempted. Rapidly developing technology for deep brain stimulation^66^ may afford more selective and effective tools to explore in patients the hypothesis that the anterior insula may represent a target for treatment of AUD. Finally, it should be noted that very recent preclinical evidence in a rat model of AUD demonstrates reduction in alcohol consumption after excitatory, but not inhibitory, stimulation of the anterior insula by DREADDs.^55^ This suggests that clinical effects in patients may strongly depend on the specific stimulation protocol applied.The present study presents several limitations. Firstly, although our data set was obtained from a well‐characterized cohort of AUD patients and healthy subjects,^26^ the group size was relatively small (albeit in the range of other rsfMRI studies), and only male participants were included. Thus, sex effects on the observed connectivity alterations need to be investigated more closely in the future.

Secondly, the AUD group in this study contained a significantly higher number of smokers compared with controls. Thus, we cannot exclude nicotine as a contributing factor to the observed differences in modular structure of brain networks in AUD patients and healthy subjects. However, we note that no correlation was found within the patient group between network parameters (participation coefficient, global efficiency, etc.) and smoking variables. Moreover, patients were allowed to smoke ad libitum during the study and the subsequent observation period. Hence, changes in modularity over the 2‐week observation period argues against smoking as a driver of the effects on modularity we have observed.

Thirdly, subjects included in this study were selected to be free of psychiatric comorbidities but showed subclinical anxiety or depression symptom scores, very often observed in AUC patients.

We note that depression and anxiety disorders have been associated with some abnormal connectivity. However, because these scores were not clinically relevant, and their relations with exposure and graph‐related outcome measures are unknown, they were not accounted for in our analytical design.

Finally, in our exploratory analysis of the stability of topological changes in functional connectivity, we used data obtained from a naturalistic open‐label trial,^26^ which limits the inference that can be made from this study. We opted for an open‐label design over the gold‐standard randomized control trial (RCT) because the NTX option was already implemented in our standardized treatment programme.^29^ Thus, the allocation of patients to NTX treatment based on informed choice in the framework of an open‐label study encourages compliance and represents a more accurate picture of clinical practice. Anyway, patients in the two subgroups did not differ in clinical baseline characteristics and treatment outcome, and we found largely the same changes on network topology. Therefore, we believe that the naturalistic design did not invalidate our conclusions of partially recovered network topology especially of insula connectivity by prolonged abstinence.

## 5 CONCLUSION

In conclusion, we have studied the modular organization of resting‐state functional connectivity in a cohort of recently detoxified alcoholics and in matched healthy controls. We observed overall widespread reduction in the functional connectivity of patients. However, the effects of these alterations on the organization of functional connectivity were region specific and involved the supramarginal and the basal modules, resulting in the break‐up of these clusters. A significantly different topological role was observed for the anterior insula, whose centrality appeared to be stronger in patients. This is consistent with the idea that the anterior insula, a critical region for the relay of interoceptive states into emotional and decision‐making processes, may play an exaggerated integrative role in AUD patients. After 2 weeks of treatment and continued abstinence, some of these effects were reversed, with a significant decrease of centrality of the insula.

These observations were the direct result of recent methodological advancements in graph‐theoretical analysis that made it possible to analyze and compare the modular organization of functional connectivity in patients and controls at a finer scale and with less bias than previously possible. This study paves the way to the extension of these approaches to other neuropsychiatric conditions.

## CONFLICT OF INTEREST

DH received honoraria from Indivior, Camrus and Servier for participation in advisory boards on matter unrelated to the work presented here. The other authors have no financial interests nor other conflicts of interest to disclose.

## Supporting information


**Data S1.** Supporting Information

## Data Availability

The data that support the findings of this study are available on request from the corresponding author. The data are not publicly available due to privacy or ethical restrictions.
