# Supplementary material for: Increased network centrality of the anterior insula in early abstinence from alcohol
Source: Addict Biol. 2021 Aug 31;27(1):e13096. doi: 10.1111/adb.13096 (PMC9286046; doi:10.1111/adb.13096)
Supplement: Supplementary file 1 — Data S1. Supporting Information [file ADB-27-0-s001.docx]

**Supplementary Information**

**Extended Materials and Methods**

*Participants*

The experimental groups consisted of 35 male, recently detoxified, abstinent alcoholics (age= 45±9, abstinence days 21±7, 260±120 [g]/day of alcohol pre-treatment) and 34 healthy male volunteers (age= 41±10) recruited within the ERA-NET NEURON TRANSCALC study (WHO-International Clinical Trials Registry Platform: DRKS00003357). Clinical characteristics are listed in Table 1. The key inclusion criteria for the AUD group was an AUD diagnosis according to DSM-IV, controlled abstinence of at least 2 weeks prior to the MRI session and completion of medically supervised detoxification (treatment of withdrawal symptoms with short-acting benzodiazepines had to be completed for at least 3 days, i.e., > 5× elimination half-life [t½]). Patients with psychiatric comorbidities were excluded, as well as subjects with abuse of other substances, with the exception of smoking (all subjects underwent urine test prior to admission). All patients participated in an Intensive Withdrawal Treatment (IWT), that includes occupational therapy, physical activation, psychoeducation, psychological group therapy and psychological one-on-one sessions as well as multiple medical rounds (see (1)). The study was approved by the ethics committee of the Faculty of Medicine Mannheim of the University of Heidelberg. All participants provided written informed consent according to the Declaration of Helsinki (2).

After baseline assessment, patients were offered the choice between treatment as usual, that is continued IWT, or IWT plus adjuvant oral Naltrexone (NTX, 50mg per day) in a naturalistic open-label free-choice design. 29 patients were included in this arm of the study, with 12 subjects receiving IWT only, and 17 IWT+NTX (Tab.2). Daily intake of NTX was supervised during inpatient treatment to ensure adherence to medication. After discharge, adherence was monitored during the follow-up interviews by patient report and monitoring of prescription frequency. A follow-up fMRI scan was scheduled for all patients two weeks into treatment with either NTX plus treatment as usual or ITW only (M = 15.5 days, SD = 3.5).

The study was approved by the ethics committee of the Faculty of Medicine Mannheim of the University of Heidelberg. All participants provided written informed consent according to the Declaration of Helsinki (2).

*Clinical assessment*

Only right-handed subjects (handedness laterality quotient according to the Edinburgh Handedness Inventory (3)greater than 50) with a normal or corrected-to-normal vision (binocular visual acuity ≥ .8) were included. All participants were assessed by Structured Clinical Interview for DSM-IV to identify patients who met criteria for AUD; exclude subjects who met criteria for Axis I or II psychiatric disorder within the past 12 months (except alcohol or nicotine dependence), had current use of psychotropic or anticonvulsive medication, had positive urine drug screening (opiates, cannabinoids, benzodiazepines, barbiturates, cocaine, amphetamines), or unstable medical conditions. Quantity of lifetime and recent alcohol consumption and date of last drink were obtained by interview (4). All participants completed questionnaires as detailed in tab.1. Clinical characteristics of the subjects subsequently treated with ITW or ITW+NTX are reported in tab.2.

Drinking and relapse data were collected for three months following the experiment using the Form 90 (5). Relapse was further defined as relapse to heavy drinking if patients’ alcohol consumption exceeded 48 grams per day for women and 60 grams per day for men. In accordance with our earlier studies, we used time to relapse to heavy drinking as outcome variable in our survival analyses (6).

*MRI Acquisition and Preprocessing*

Scanning was performed with a 3 T whole-body tomograph (MAGNETOM Trio with TIM technology; Siemens, Erlangen, Germany). T2* weighted echo-planar images (EPI) were acquired in a transversal orientation 30° clockwise to AC-PC-line covering the whole brain with the following parameters: TR=1.5 s, TE=28 ms, flip angle = 80°, 24 slices, slice thickness 4 mm, 1 mm gap, voxel dimensions 3 x 3 x 5 mm^3, FOV 192x192 mm^2, 64x64 in-plane resolution. This short TE and the 30° flip to AC-PC orientation were chosen to minimize susceptibility artifacts. The number of images measured for each subject was 240. In addition, a T1-weighted 3D MPRAGE dataset consisting of 192 sagittal slices (slice thickness 1 mm, 1×1×1 mm voxel size, FOV 256 x 256 mm^2, TR= 2300ms, TE = 3.03 ms, TI = 900 ms, flip angle = 9°) was acquired. Physiological data were acquired with the SIEMENS standard sensors for pulse oximetry and respiration with a sample rate of 50 Hz. During the resting-state scan participants were instructed to close their eyes and not to think of something specific, to let their mind wander and not to hold on to their thoughts.

The fMRI data were preprocessed using SPM8 (Welcome Trust Centre for NeuroImaging, London, UK) and FSL (v. 5.0). After discarding the initial 10 volumes of each participant, the remaining volumes were processed to remove physiological confounds (heartbeat, respiration) from the raw data using the Aztec toolbox (7). The resulting corrected volumes were slice-time corrected, head-motion realigned (patients average maximum translation = 0.545±0.465, average maximum rotation= 0.007±0.006; control average maximum translation = 0.502±0.474, average maximum rotation= 0.007±0.007) and normalized to the standard MNI EPI template space (voxel-size resampled to 3x3x3mm^3^). The signal related to head movement was removed using a multiple regression model.

Additionally, Framewise Displacement (FD) and DVARS, two indices of data quality, were computed following (8). FD is a measure of head motion between subsequent volumes, whereas DVARS is an index of signal intensity changes across volumes. No outliers were identified in either group, nor significant between-group differences were observed in these metrics, and in the number and extent of motion-related spikes (tab.S4 and S5). Comparisons of motion parameters, DVARS and FD for the experimental groups are reported in tab.S4, S5, fig.S1, S2. In the light of this analysis we did not apply frame censoring to avoid loss of information and decrease of temporal degrees of freedom (9, 10). Potentially residual spurious correlations were removed at the level of correlation networks following (38) (see below for further detail).

*Connectivity graphs*

The template of Crossley et al. (11) was used to parcellate the whole brain of each participant into 638 cortical and subcortical regions of interest, each representing a node in the network. This atlas, based on functional parcellation, has been previously applied to the study of other psychiatric disorders, like schizophrenia (12). It includes a relatively large, yet computationally affordable number of parcels, which show relatively uniform size distribution, and were defined on the basis of fMRI driven functional criteria. Blood Oxygenation Level Dependent (BOLD) time series were extracted and averaged for every node, and Pearson correlation coefficients were calculated for all pairs of nodes, thus providing an adjacency matrix for each subject in the study. Group-level functional connectivity matrices were computed by Fisher-transforming and subsequent averaging of individual’s adjacency matrices and subsequently sparsified by percolation analysis (13). Sparsification procedures are often applied to remove the weakest edges, which are the most affected by experimental noise and likely to contain spurious correlations. This procedure, initially introduced by Gallos et al (2012) (13), iteratively removes the weakest edges and computes the network’s largest connected component. The procedure stops when the largest component starts breaking apart to identify a threshold that preserves network structure and connectedness while removing potentially spurious correlations. We have recently shown that the percolation threshold maximizes information extracted by the subsequent application of community detection algorithms (14), and applied and validated the method in human (15) and animal (16) studies, as well as in synthetic networks (14). Moreover, we have demonstrated that percolation analysis can effectively mitigate the deleterious effects of motion-related correlations on functional connectivity networks and maximize detectability of large-scale network structures, which may otherwise be erased by excessively aggressive motion-correction approaches (38), thus preventing detection of inter-group differences. In summary, potentially spurious correlations were controlled by a three-pronged approach: 1) careful selection and matching of patients and controls based on two different motion-sensitive metrics (FD and DVARS); 2) recording of physiological signals and removal by AZTEC toolbox; 3) sparsification of resulting adjacency network by percolation analysis.

Percolation analysis was performed independently in the two groups prior to community detection. This represents an important difference from previous studies investigating community structure in functional connectivity networks in patients and controls. Typically, thresholds are determined by fixing the same edge densities in the connectivity graphs of the groups under investigation to enable comparison of density-dependent measures of connectivity. However, imposing equal densities to graphs describing connectivity in groups with different connectivity strength, like in the present case, may lead to the inclusion of a greater number of potentially spurious links in the group with weaker connectivity, and to the exclusion of important links in the group with stronger connectivity. A higher proportion of spurious connection results in a more random network topology, and intergroup differences may just reflect different levels of noise, rather than genuine topological differences (17). Community structure is defined on the basis of membership of nodes in different clusters, and does not depend on edge density, but on the balance between intra- and inter-cluster edges. Hence, identification that maximizes information about the modular organization of each of the networks to be compared enables unbiased comparison between groups, as demonstrated by (14). Here, the optimal sparsification thresholds were determined independently in the patient and control to enable unbiased comparison of the respective community structures (12).

*Network metrics*

From the adjacency matrix, we extracted the distribution of z-score (corresponding to the weighted edges of our network), and we computed nodal and global measures of connectivity. The degree of a node represents the number of connections towards other nodes.

The network density indicates the ratio between the connections in the matrix after sparsification and all possible connections (18).

Global efficiency can be interpreted as a measure of how efficiently information is exchanged across the network (19). It is defined as the inverse of the harmonic mean of the shortest weighted path lengths connecting every pair of nodes, and is inversely related to the network characteristic path length (20). The weighted global efficiency can be written as:

$$E_{glob}\left( G \right)=\frac{1}{n(n-1)}\sum_{i<j} \frac{1}{d_{ij}^{w}}$$

where $d_{ij}^{w}$ is the weighted shortest-path length between node *i* and node *j* of a graph with *n* nodes. The weighted path-length represents the number of edges that separate two nodes, each weighted by the edge weight.

Local efficiency is defined as the efficiency of a local subgraph consisting of a node *i*’s nearest neighbors, excluding the node *i* itself, and quantifies a network’s resistance to failure on a local scale. The definition of weighted local efficiency used in this manuscript is the one given by (20):

$$E_{loc}^{w}=\frac{1}{n}\sum_{i\in N} \frac{\sum_{j,h\in N, j\neq i} \left( w_{ij}w_{ih}\left[ d_{jh}^{w}\left( N_{i} \right) \right]^{-1} \right)^{\frac{1}{3}}}{k_{i}(k_{i}-1)}$$

where N is the set of all nodes in the network and n is the number of nodes.

*Modular organization by InfoMap*

The most widely applied method for community detection in brain connectivity graphs is Newman’s Modularity (21), implemented in the Brain Connectivity Toolbox (20). However, it has been recently shown that this method, alongside with most others, suffers from two fundamental problems (22, 23). Firstly, degeneracy of nearly-optimal solutions, whereby similar values of the fitness function around its maximum correspond to substantially different partitions (24). Secondly, Newman’s modularity is unable to resolve modules that are smaller than a scale determined by the size of the entire network, a shortfall sometimes referred to as the “resolution limit” (25).

Here, we have applied InfoMap, a method based on the optimization of a cost function dubbed map equation (26, 27). We have recently shown that this method is superior to Newman’s Modularity in terms of sensitivity and specificity in the presence of heterogeneously distributed modules, and overcomes some of the fundamental limitations of Newman’s Modularity (23). Moreover, InfoMap has been widely applied and validated in community detection studies in natural networks, including brain connectivity networks from clinical studies involving neuropsychiatric patients (28). The idea behind Infomap is the minimization of the description length (29) of a random walker defined on the network through a set of heuristics. Here we used the Infomap implementation for weighted networks available in the igraph-0.7.1 package (30).

In order to generate a stable solution from a non-deterministic method like InfoMap, we applied a consensus approach (31). This method provides a means to compute a stable partition that is representative of the consensus of all nearly-optimal solutions generated by different runs of the community detection algorithm. Its application consists of two steps. First, the generation of the consensus matrix (n x n, with n the number of nodes), which is obtained by running the community detection InfoMap method 1000 times and assigning a value to the element t_ij_ of the matrix that corresponds to the number of times nodes i and j appear in the same community. The second step involves running the community detection algorithm (InfoMap, in our case) on the consensus matrix to generate the consensus partition. The computation of the consensus partition was performed using a modified version of the function provided by the Brain Connectivity Toolbox (20) adapted to weighted networks.

All visual representations of the anatomical distribution of modules and topological parameters were produced using the BrainNet viewer toolbox (32) and MRIcron (33).

*Network Based Statistics*

To evaluate edge level statistical differences between groups the Network Based Statistics (NBS) toolbox was used (34). NBS performs a mass-univariate testing on all graph edges, controlling for the Family-Wise Error Rate (FWER), in order to overcome the multiple comparison problem when testing differences across every connection. A statistical threshold was selected at a *t* value of 3.1; next, a t-test was applied to run 5000 permutations on all the connections, comparing the identified supra-threshold components across groups. Given the overall weakening of functional connectivity in the patient group, the contrast was set in order to detect weaker connections in the patient group. This analysis was covaried for age.

*Group level comparison of modular organization.*

After community detection by InfoMap in the two experimental groups (patients and controls), we computed the similarity between the extracted modular partitions in terms of normalized mutual information (NMI) (35), a parameter rooted in Information Theory. Specifically, we used an approach proposed in (36) to test for statistical differences between groups. This statistical method is based on the idea that if variance in the community structure data is reliably explained by group membership, then the mean NMI between all possible pairs of participants within an experimental group should be higher than the mean NMI of pairs of participants from random groups. Since the distribution of group means NMI is not known *a priori*, a null-distribution is generated through a permutation method between the two experimental groups (10000 permutations).

The same analysis was applied in the comparison between the pre- and post-treatment resting state data in the group of patients, and in the two subgroups (IWT only or IWT+NTX).

*Participation coefficient*

To complete the investigation at the node level, we considered the alteration in node role between the two populations based on the differences in modular organization. To this end, we adopted Guimera’ and Amaral classification scheme (37) , whereby nodes are classified by their within-community degree (a measure of how well connected a node is to other nodes in the same community) and their participation coefficient *P*, a parameter that reflects the extent to which a node is connected to nodes in other modules. *P* can be written as:

$$P_{i}=1-\sum_{c=1}^{N_{M}} \left( \frac{k_{ic}}{k_{i}} \right)^{2}$$

where $k_{ic}$ is the number of links of node *i* to nodes in module *c*, and $k_{i}$ is the total degree of node *i*. To assess statistical differences, we computed the *P* index of each node for each subjects and ran a t-test between groups, Bonferroni corrected. Node-wise statistical significance was parametrically mapped on the MRI template. Nodes with high Participation Coefficient are characterized by high centrality and are important for the integration of various modules into a cohesive, efficient network structure (37).

*Post-hoc correlations with clinical variables*

To test if clinical variables could predict alterations in global efficiency metrics or for selected participation coefficients (see results) we used multiple linear forced entry regression analysis (IBM SPSS statistics software version 20, IBM Corp., Armonk, NY, USA) with a model comprising alcohol consumption (standard drinks containing 12 g alcohol per drinking day) and severity of alcohol dependence (ADS) as well as age as control variable. Likewise, the effect of smoking was tested including the variables age, FTND and pack years.

Follow-up relapse data and neural connectivity data was available for 17 NTX patients and 10 patients receiving standard treatment (N = 27). Cox regression models were implemented to test the main effect of naltrexone on time to first severe relapse, as well as associations between relapse risk and local and global connectivity measures (i.e. global efficiency and local efficiency and participation coefficients of seven insular nodes) and the interaction of both factors.

**Summary of subjects’ data**

**Table S1:** Demographic and clinical data for healthy controls and patients

|  | **Healthy subjects** | **Alcohol dependent patients** |
| --- | --- | --- |
| Total N (fMRI data available) | 34 | 35 |
| Age | 41.35 (10.00) | 45.75 (8.96)^+^ |
| AUDIT | 2.82 (2.25) | 26.67 (6.53)** |
| ADS | 2.16 (2.44) | 14.08 (6.55)** |
| Duration of alcohol dependence (years) |  | 13.38 (10.55) |
| *Alcohol consumption* |  |  |
| Drinks/day in the last 90 days | 3.08 (1.73) | 21.64 (10.98)**^#^** |
| Number of drinking days in last 90 days | 17.46 (19.24) | 77.53 (20.14)^#^ |
| Abstinence before fMRI (days) | 14.14 (37.92) | 23.71 (17.14)** |
| *Alcohol craving* |  |  |
| OCDS | 1.37 (1.25) | 17.66 (6.78)** |
| CAS-A non-intentional | 1.15 (2.84) | 22.59(15.65)** |
| CAS-A non-volitional | 1.00 (2.59) | 31.85 (14.14)** |
| *Smoking* |  |  |
| N (current smoker) | 4 | 30^§^ |
| FTND (in smokers) | 4.75 (3.95) | 6.31 (2.33) |
| *Depression and anxiety symptoms* |  |  |
| BDI | 2.03 (2.65) | 12.16(8.93)** |
| STAI State | 31.94 (5.60) | 39.32 (11.28)* |
| STAI Trait | 30.52 (7.34) | 41.94 (10.78)** |

A standard drink corresponds to 12 g alcohol. t-test: ^+^p =.057, *p<.01, ** p< .001; Mann-Whitney U test: ^#^p< .001, Chi-square test: ^§^p<.05. AUDIT - Alcohol Use Disorders Test (Saunders *et al.*, 1993); ADS – Alcohol Dependence Scale (Kivlahan *et al.*, 1989); OCDS - Obsessive Compulsive Drinking Scale; CAS-A - Craving Automated Scale for Alcohol (Vollstädt-Klein *et al.*, 2015); FTND - Fagerstrøm Test for Nicotine Dependence (Fagerstrom and Schneider, 1989); BDI - Beck Depression Inventory (Beck *et al.*, 1961); STAI - State-Trait Anxiety Inventory(Spielberger, 1983).

**Table S2:** Clinical characteristics for patient groups with available imaging data for both time points (baseline and week two scan)

|  | **No medication** | **NTX** |
| --- | --- | --- |
| Total N (fMRI data available for baseline and follow-up) | 12 | 17 |
| Age | 44.75 (8.38) | 49.88 (6.34) |
| AUDIT | 28.86 (7.19) | 23.88 (6.98) |
| ADS | 15.19 (6.61) | 13.13 (5.89) |
| Duration of alcohol dependence (years) | 16.27(12.17) | 15.29 (10.47) |
| *Alcohol consumption* |  |  |
| Drinks/day in the last 90 days | 20.03 (14.65) | 16.25 (11.68) |
| Number of drinking days in last 90 days | 75.42 (25.89) | 77.12 (19.41) |
| Abstinence before fMRI (days) | 21.36 (6.96) | 26.76 (23.28) |
| OCDS | 17.08 (8.14) | 14.88 (6.21) |
| CAS-A non-intentional | 28.36 (14.38) | 16.80 (14.89) |
| CAS-A non-volitional | 36.45 (10.51) | 23.44 (14.93)* |
| *Smoking* |  |  |
| N (current smoker) | 11 | 13 |
| FTND (in smokers) | 6.17 (2.55) | 5.00 (2.94) |
| *Depression and anxiety symptoms* |  |  |
| BDI | 38.58 (12.16) | 38.63 (9.18) |
| STAI State | 41.42 (10.41) | 39.00 (12.35) |
| STAI Trait | 20.03 (14.65) | 16.25 (11.68) |

A standard drink corresponds to 12 g alcohol. T-test: * p < 0.05. 1. AUDIT - Alcohol Use Disorders Test (Saunders *et al.*, 1993); ADS – Alcohol Dependence Scale (Kivlahan *et al.*, 1989); OCDS - Obsessive Compulsive Drinking Scale; CAS-A - Craving Automated Scale for Alcohol (Vollstädt-Klein *et al.*, 2015); FTND - Fagerstrøm Test for Nicotine Dependence (Fagerstrom and Schneider, 1989); BDI - Beck Depression Inventory (Beck *et al.*, 1961); STAI - State-Trait Anxiety Inventory(Spielberger, 1983).

**Global connectivity parameters**

**Table S3:** Connectivity metrics for the two experimental groups.

|  | Number of edges | Density | Average Degree | Global efficiency | Average local efficiency |
| --- | --- | --- | --- | --- | --- |
| Control | 17390 | 0.35 ±0.22 | 222.74 ±143.39 | 0.43±0.13 | 0.62±0.10 |
| AUD | 9356 | 0.21 ±0.14 | 130.97 ±91.91 | 0.39±0.08 | 0.64±0.08 |
| p-value | 0.004 | 0.004 | 0.002 | 0.014 | 0.55 |

**Assessment of motion in the patient and control group**

**Table S4:** Summary statistics of FD and DVARS between-group comparison

|  | Healthy Controls | AUD | Statistics |
| --- | --- | --- | --- |
| Framewise Displacement |  |  | t=1.291 , p=0.201 |
| Mean | 0.2612 ± 0.1355 | 0.3012 ± 0.1217 |  |
| Min. | 0.1085 | 0.1436 |  |
| Max. | 0.7756 | 0.7067 |  |
| DVARS |  |  | t=0.960, p=0.340 |
| Mean | 13.9663 ± 3.9780 | 14.7943 ± 3.1465 |  |
| Min. | 9.4933 | 10.6499 |  |
| Max. | 28.3804 | 23.3897 |  |

**Table S5:** Mean of the number of volumes resulting to be outliers according to FD and DVARS metrics, with the percentage of affected scan time.

|  | HC | ADP | T test (t, p) |
| --- | --- | --- | --- |
| FD | 5.53±4.94 (2.3%) | 5.69±7.62 (2.5%) | 0.214, 0.831 |
| DVARS | 10.88±4.54 (4,7%) | 11.08±6.46 (4.8%) | 0.151, 0.880 |

**Framewise Displacement**


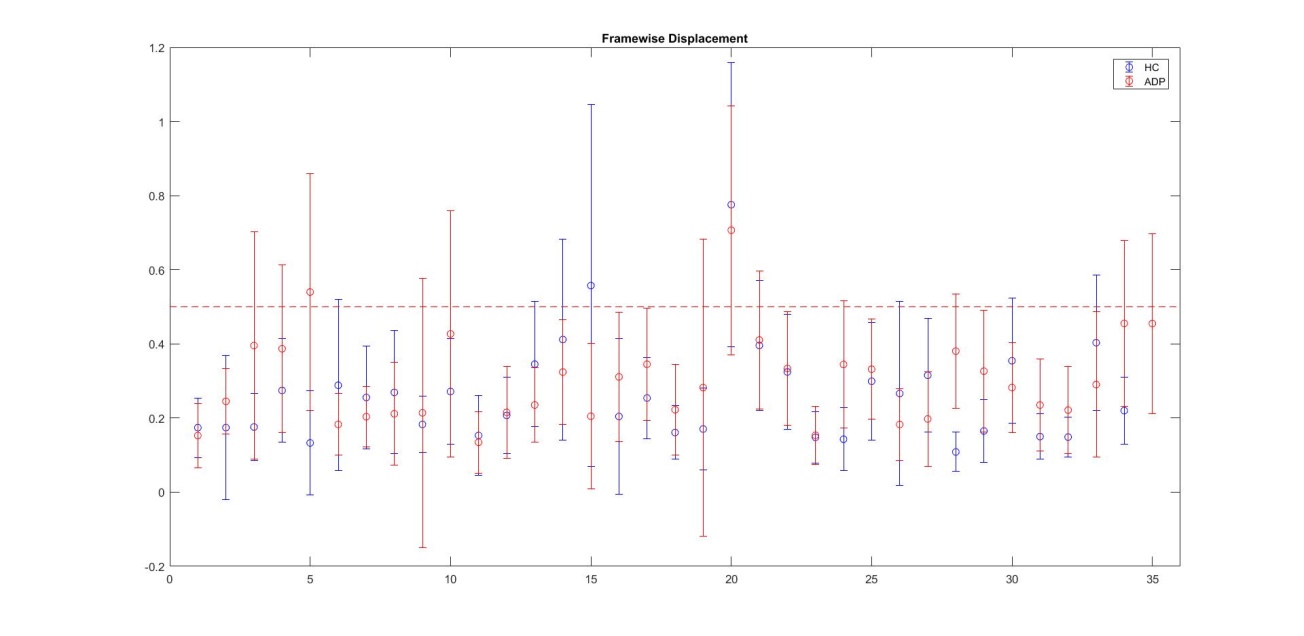


**Figure S1:** Comparison of Framewise Displacement in AUD patients and healthy controls, in red and blue, respectively; no statistically significant difference was observed between the two groups (See Tab S1 and S2).

**DVARS**


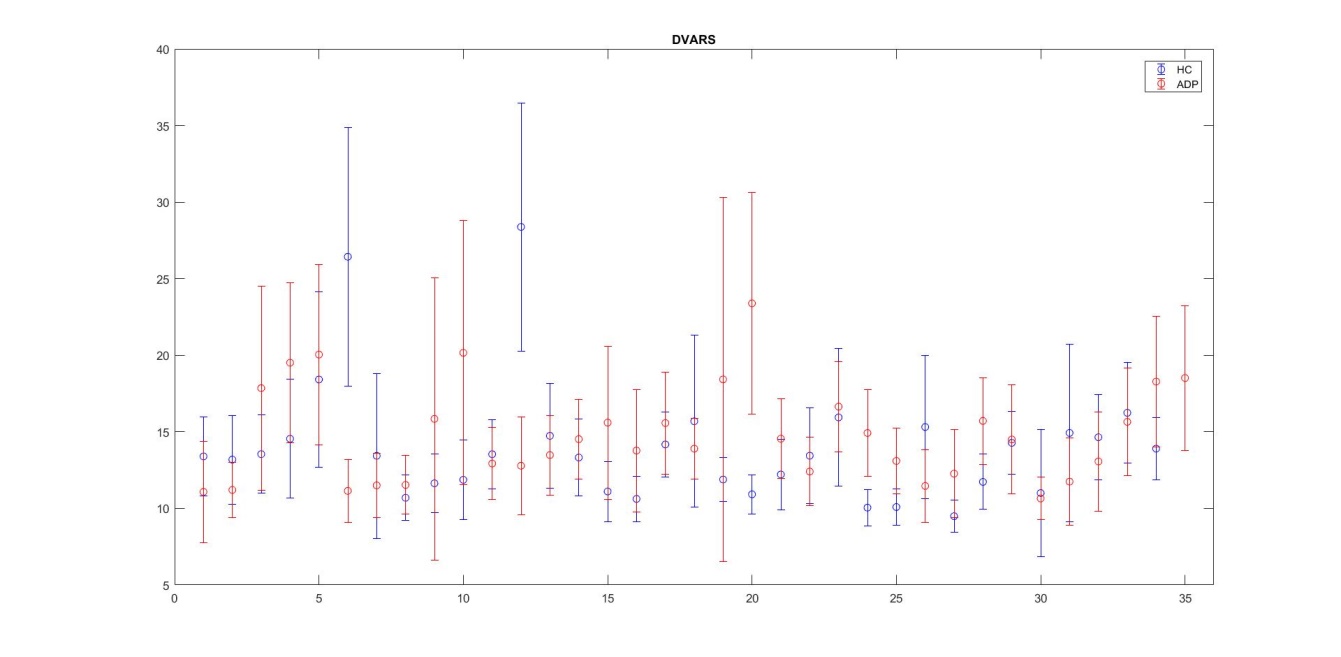


**Figure S2**: Comparison of DVARS in AUD patients and healthy controls, in red and blue, respectively; no statistically significant difference was observed between the two groups (see Tab. S1 and S2).

## Anatomical/functional identification of the 15 biggest modules of each population

 **Table S6.**

**Between-group Network-Based Statistics**


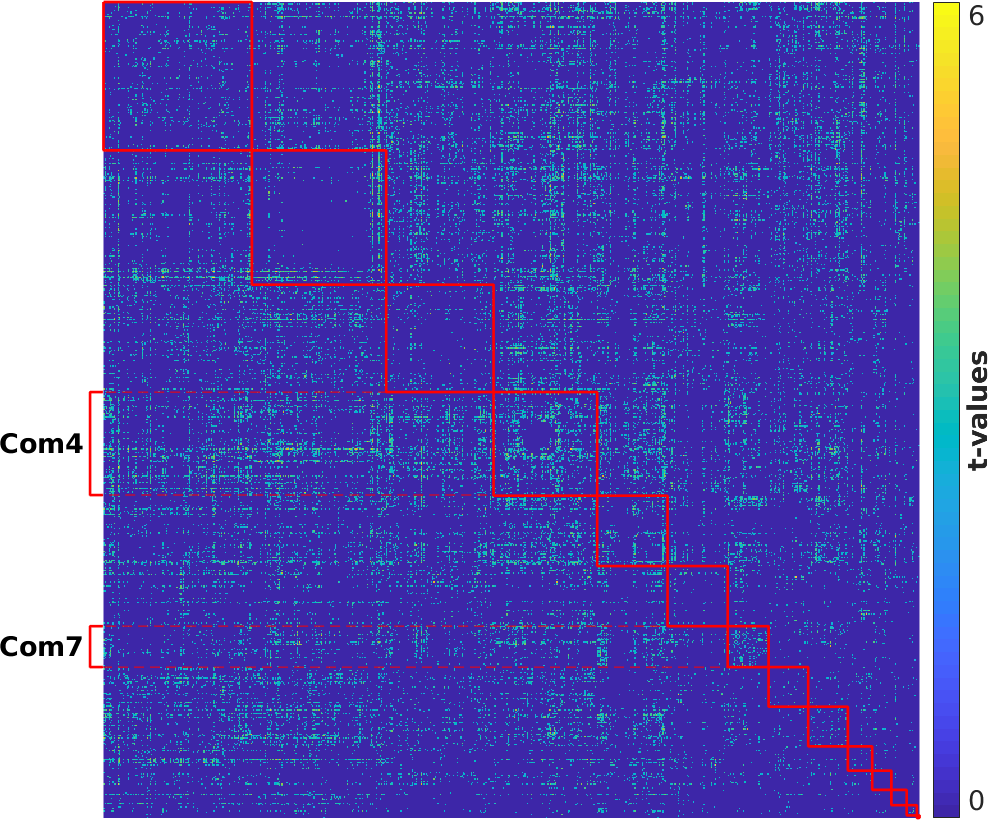
**Figure S3**: Network Based Statistics of edge strength differences between the two groups. Nodes have been reordered to reflect the modular structure in the healthy control group (modules delineated in red, with increasing size left to right). With a liberal t-value > 2.5, and testing for significantly weaker links in patients versus controls, NBS analysis identified 19058 significantly weaker connections implicating 627 different nodes (over the original 638). Significant differences in the connections between modules (the matrix elements outside the red boxes) appear to be distributed relatively evenly across the network. Conversely, differences in intramodular connections are mostly observed in specific modules. Modules 4 and 7, corresponding to the Supramarginal and Basal Modules, respectively, show over 50% of significantly weaker intramodular edges.

**Effects of treatment on brain functional connectivity networks in AUD patients**

**
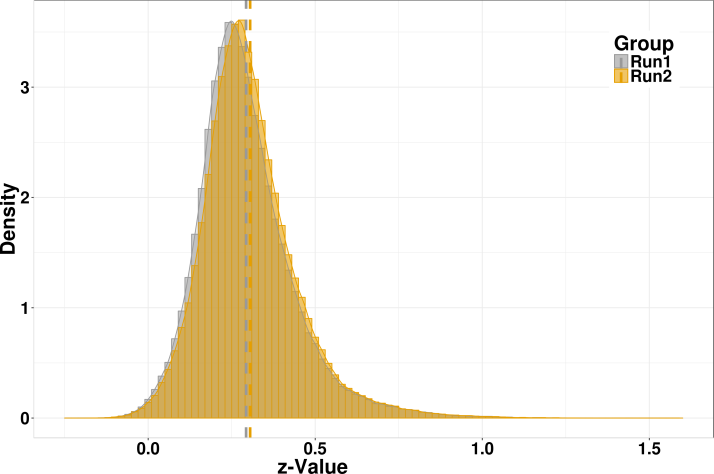
**

**Figure S4**: Z-value distributions for the adjacency matrices at baseline and after 2 weeks of treatment (Run 1 and Run 2, in grey and yellow, respectively); a right shift in the distribution indicates stronger connectivity after treatment, with a small but significant effect (p=7.4x10^-04^).

**Participation Coefficient Insula**

**
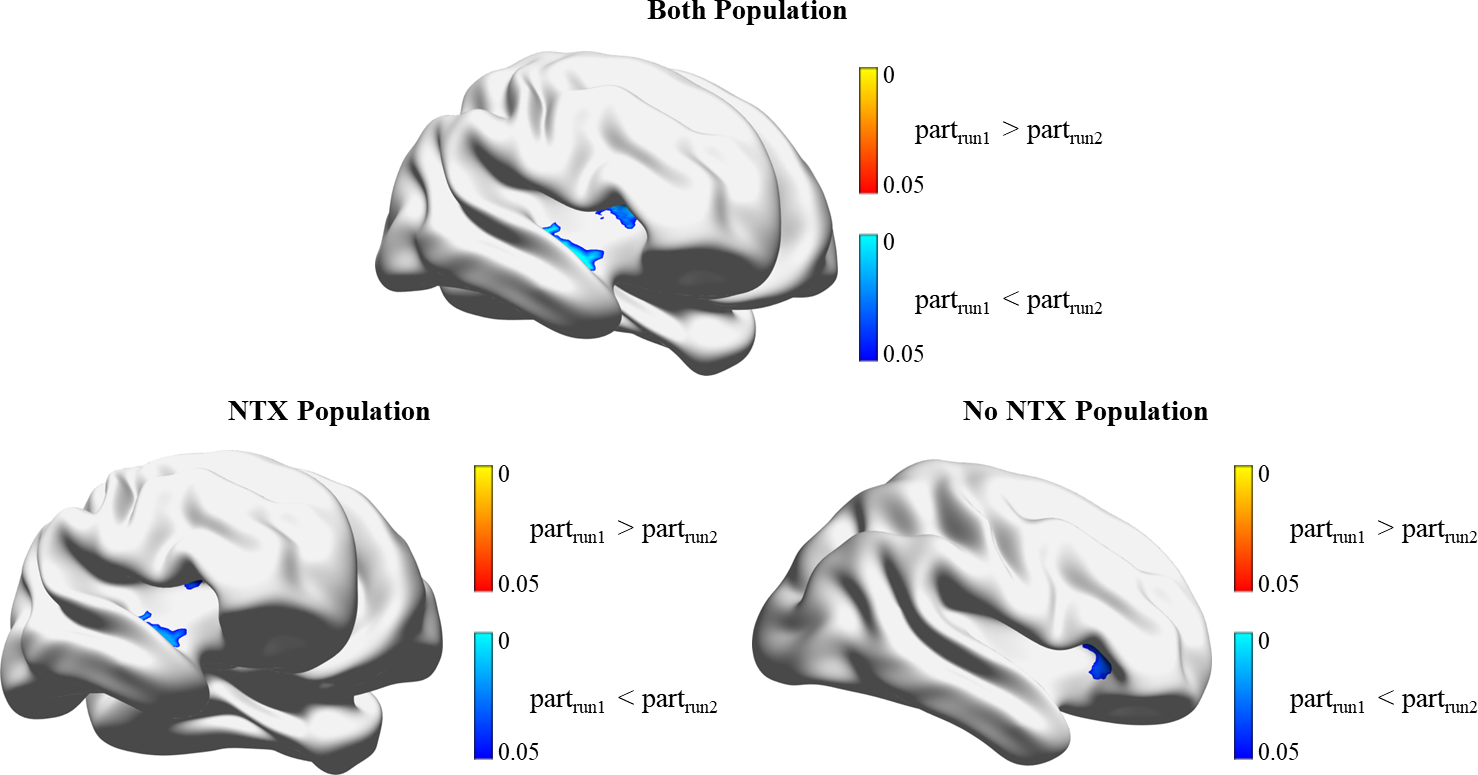
**

Figure S5: Map of differences in participation coefficient pre and post treatment. Projections of the p-values obtained by a one-tailed Student’s t-test, are shown comparing node-wise differences between runs, with the hypothesis of larger participation coefficient for Run1 ( before treatment) or for the Run2 (after treatment) in the top and bottom panel, respectively.

**Voxel Based Morphometry**

All the images were acquired in the Institute for Psychopharmacology from Mannheim, Germany, using a Siemens MAGNETOM Trio a Tim 3T scanner (Siemens AG, Munich, Germany). The T1-weighted MRI images consisted in 192 sagittal slices per subject (matrix size = 256 • 256, in-plane resolution = 1 • 1 mm2, slice thickness = 1 mm), TE/TR = 3.03/2300 ms, flip angle α = 9º.

The structural images were analyzed with FSL-VBM.

*Preprocessing*

Three steps were required before performing the VBM: 1) brain segmentation, 2) study-specific GM template creation and 3) registration of the native GM images. All the structural images were brain-extracted using BET (Brain Extraction tool) and gray matter-segmented employing FAST (FMRIB’s Automated Segmentation Tool). The two groups under study, AUD patients and healty controls, had to be equally represented in the study-specific GM template to avoid any bias during the registration step. That is, if one group had a greater number of GM images selected for creating the template, the non-linear registration may have been more accurate for that group, leading to registration-related differences in the GM volume distribution. Therefore, eighteen controls and eighteen patients were selected to create the GM template. The template-selected GM images were affine-registered to the GM ICBM-152 template, concatenated and averaged. The resulted 4D image was then flipped along the x-axis to create a left-right symmetric template and the two mirror images were then re-averaged to obtain a first-pass GM template. Subsequently, the template-selected GM images were re-registered to this first-pass GM template using non-linear registration, concatenated into a 4D image, averaged and flipped along the x-axis. Both mirror images were then averaged to create the final symmetric, probabilistic and study-specific GM template in standard space at 2 • 2 • 2 mm3 resolution. 3.

Finally, all the GM images were non-linearly registered (free-form deformation with 20 mm initial control point spacing) to the study-specific template and concatenated into a 4D image. There was a modulation (compensation) to correct for local enlargement or contraction due to the non-linear registration: each voxel of each registered GM image was multiplied by the Jacobian of the warp field. The last steps in the preprocessing were the concatenation of the modulated registered GM images into a 4D image and its smoothing by a an isotropic Gaussian kernel with a sigma of 3.5 mm (8mm FWHM, full with at half maximum).

*Analysis*

Permutation-based non-parametric inference, correcting for multiple comparisons across space, was applied in the unpaired study using the design matrix and contrasts showed in fig.S7, a GM mask and the 4D multi-subject preprocessed data. Threshold-free cluster enhancement (TFCE) approach was employed to enhance the intensity within cluster-like regions more than background (noise) regions, making it easy to find a threshold that discriminated between noise and spatially-extended signal. The contrast with the hypothesis that GM is greater in healthy controls than in abstinent alcoholics is shown in fig.S6. No significant effects were observed in the Insula nor in the Amygdala.


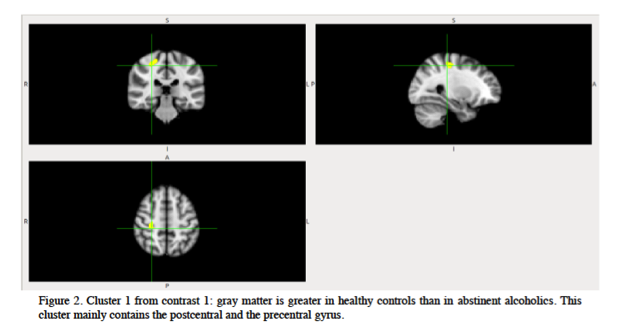


**Figure S6:** **Cluster from contrast: gray matter is greater in healthy controls than in abstinent alcoholics.** This cluster mainly contains the postcentral and the precentral gyrus. No significant differences were observed in the Amygdala or in the Insular Cortex.

**References**

1. Loeber S, Kiefer F, Wagner F, Mann K, Croissant B (2009) Behandlungserfolg nach qualifiziertem Alkoholentzug. *Nervenarzt* 80(9):1085–1092.

2. Rickham (1964) World Medical Association. Code of Ethics of the World Medical Association. Declaration of Helsinki. *Br Med J*.

3. Oldfield RC (1971) The assessment and analysis of handedness: The Edinburgh inventory. *Neuropsychologia* 9(1):97–113.

4. Scheurich A, et al. (2005) Reliability and validity of the form 90 interview. *Eur Addict Res*. doi:10.1159/000081417.

5. Tonigan J, Miller W, Brown J (1997) The reliability of Form 90: an instrument for assessing alcohol treatment outcome. *J Stud Alcohol*. doi:10.15288/jsa.1997.58.358.

6. Bach P, et al. (2015) Increased mesolimbic cue-reactivity in carriers of the mu-opioid-receptor gene OPRM1 A118G polymorphism predicts drinking outcome: A functional imaging study in alcohol dependent subjects. *Eur Neuropsychopharmacol*. doi:10.1016/j.euroneuro.2015.04.013.

7. Van Buuren M, et al. (2009) Cardiorespiratory effects on default-mode network activity as measured with fMRI. *Hum Brain Mapp* 30(9):3031–3042.

8. Power JD, Barnes KA, Snyder AZ, Schlaggar BL, Petersen SE (2012) Spurious but systematic correlations in functional connectivity MRI networks arise from subject motion. *Neuroimage* 59(3):2142–2154.

9. Ciric R, et al. (2017) Benchmarking of participant-level confound regression strategies for the control of motion artifact in studies of functional connectivity. *Neuroimage*. doi:10.1016/j.neuroimage.2017.03.020.

10. Bright MG, Tench CR, Murphy K (2017) Potential pitfalls when denoising resting state fMRI data using nuisance regression. *Neuroimage*. doi:10.1016/j.neuroimage.2016.12.027.

11. Crossley NA, et al. (2013) Cognitive relevance of the community structure of the human brain functional coactivation network. *Proc Natl Acad Sci* 110(28):11583–11588.

12. Bordier C, Nicolini C, Forcellini G, Bifone A (2018) Disrupted modular organization of primary sensory brain areas in schizophrenia. *NeuroImage Clin* 18:682–693.

13. Gallos LK, Makse HA, Sigman M (2012) A small world of weak ties provides optimal global integration of self-similar modules in functional brain networks. *Proc Natl Acad Sci U S A* 109(8):2825–2830.

14. Bordier C, Nicolini C, Bifone A (2017) Graph analysis and modularity of brain functional connectivity networks: Searching for the optimal threshold. *Front Neurosci* 11(AUG). doi:10.3389/fnins.2017.00441.

15. Alexander-Bloch AF, et al. (2010) Disrupted modularity and local connectivity of brain functional networks in childhood-onset schizophrenia. *Front Syst Neurosci* 4(October):147.

16. Bardella G, Bifone A, Gabrielli A, Gozzi A, Squartini T (2016) Hierarchical organization of functional connectivity in the mouse brain: a complex network approach. *Sci Rep* 6:1–11.

17. van den Heuvel M, Fornito A (2014) Brain Networks in Schizophrenia. *Neuropsychol Rev* 24(1):32–48.

18. Albert R, Barabási A-L (2002) Statistical mechanics of complex networks. *Rev Mod Phys* 74(1):47–97.

19. Latora V, Marchiori M (2001) Efficient behavior of small-world networks. *Phys Rev Lett*. doi:10.1103/PhysRevLett.87.198701.

20. Rubinov M, Sporns O (2010) Complex network measures of brain connectivity: Uses and interpretations. *Neuroimage* 52(3):1059–1069.

21. Newman ME (2006) Modularity and community structure in networks. *Proc Natl Acad Sci U S A* 103(23):8577–8582.

22. Nicolini C, Bifone A (2016) Modular structure of brain functional networks: breaking the resolution limit by Surprise. *Sci Rep* 6(19250).

23. Nicolini C, Bordier C, Bifone A (2017) Modular organization of weighted brain networks beyond the resolution limit. *Neuroimage* 146:28–39.

24. Good BH, De Montjoye YA, Clauset A (2010) Performance of modularity maximization in practical contexts. *Phys Rev E - Stat Nonlinear, Soft Matter Phys* 81(4). doi:10.1103/PhysRevE.81.046106.

25. Fortunato S, Barthélemy M (2007) Resolution limit in community detection. *Proc Natl Acad Sci* 104(1):36–41.

26. Rosvall M, Bergstrom CT (2007) An information-theoretic framework for resolving community structure in complex networks. *Proc Natl Acad Sci U S A* 104(18):7327–31.

27. Rosvall M, Bergstrom CT (2008) Maps of random walks on complex networks reveal community structure. *Proc Natl Acad Sci U S A* 105(4):1118–23.

28. Lerman-Sinkoff DB, Barch DM (2016) Network community structure alterations in adult schizophrenia: identification and localization of alterations. *NeuroImage Clin* 10:96–106.

29. Rissanen J (1978) Modeling by shortest data description. *Automatica* 14(5):465–471.

30. Csárdi G, Nepusz T (2006) The igraph software package for complex network research. *InterJournal Complex Syst* 1695:1695.

31. Lancichinetti A, Fortunato S (2012) Consensus clustering in complex networks. *Sci Rep* 2:336.

32. Xia M, Wang J, He Y (2013) BrainNet Viewer: A Network Visualization Tool for Human Brain Connectomics. *PLoS One*. doi:10.1371/journal.pone.0068910.

33. Rorden C, Brett M (2000) Stereotaxic display of brain lesions. *Behav Neurol*. doi:10.1155/2000/421719.

34. Zalesky A, Fornito A, Bullmore ET (2010) Network-based statistic: Identifying differences in brain networks. *Neuroimage* 53(4):1197–1207.

35. Danon L, Guilera AD, Duch J, Arenas A (2005) Comparing community structure identification. *J Stat Mech Theory Exp* 2005(9):P09008--09008.

36. Bloch AA, et al. (2012) The discovery of population differences in network community structure: New methods and applications to brain functional networks in schizophrenia. *Neuroimage* 59(4):3889–3900.

37. Guimerà R, Amaral LA (2005) Functional cartography of complex metabolic networks. *Nature* 433(February):895–900.

38. Nicolini C, Forcellini G, Minati L, Bifone A. Scale-resolved analysis of brain functional connectivity networks with spectral entropy. Neuroimage. 2020 Feb 7;211:116603
